# Going remote: Implementing digital research methods at an academic medical center during COVID-19

**DOI:** 10.1017/cts.2021.865

**Published:** 2021-10-06

**Authors:** Katie E. Keenoy, Eric J. Lenze, Ginger E. Nicol

**Affiliations:** 1 Washington University School of Medicine, St. Louis, MO, USA; 2 happyMedium | healthyMedium, LLC, St. Louis, MO, USA

**Keywords:** mHealth research, COVID-19, eConsent, IRB, agile project management

## Abstract

COVID-19 has forced medical research institutions to conduct clinical research remotely. Here, we describe how a university’s mHealth Research Core helped facilitate the shift to remote research during the COVID-19 pandemic. In 2019 (pre-pandemic), we conducted stakeholder interviews and leadership group sessions to identify, create, and implement resources and core functions to support investigator-initiated mHealth research. Between April 2019 and February 2020, we identified four investigator needs: 1) a **seminar series** on trends in mHealth research, 2) mHealth **case consultation** services, 3) **liaison services** with institutional regulatory compliance groups, and 4) **online navigation tools** for implementation of mHealth methods (e.g., eConsent) and for building partnerships with technology vendors. To date, the mHealth Research Core has held seven seminars, completed 71 case consultations, assisted four COVID-related clinical studies, advised the IRB on shifting to remote research, and widely disseminated eConsent navigation tools. Although pre-pandemic stakeholder and investigator needs led to the creation of the mHealth Research Core, this institutional resource played a critical role in continuing clinical research during the pandemic by assisting investigators in rapidly shifting to remote study methodology.

## Introduction

The emergence of the smartphone (iPhone, 2007 and Android, 2008) [1] was an inflection point for mobile technology, rapidly leading to the ubiquity of web-enabled mobile devices and resulting in more than 85% in the USA (in 2021) owning a smartphone and 67% of the global population being connected to mobile services (in 2019) [2,3]. Mobile technology is now employed to conduct all aspects of clinical research, such as consenting participants, conducting study assessments, and delivering treatment interventions with the aims of reducing participant burden, improving efficiency and innovation in research, and extending reach to underrepresented populations [4,5].

Prior to the SARS-CoV-2/COVID-19 pandemic, the National Institutes of Health and the National Science Foundation had both prioritized a shift towards digital methods for conducting clinical studies – to achieve efficiencies and reduce costs, as well as to improve participant access and experience [6,7].

As the world emerges from the pandemic, the scientific community finds itself in a similar situation: after witnessing transformational achievements in medical science – made possible in large part by technology – how do we continue to nurture science through innovation [8,9]? Recognizing the importance of technology in safely continuing research and clinical care efforts, private and public investment in digital health innovation rose more than 60% in 2020 [10–12]. The scientific community will benefit from continued funding opportunities and academic research institutions adopting a digital infrastructure to support investigators in using and navigating mobile technology for clinical research and healthcare delivery [4].

Here, we chronicle the pre-pandemic development of a mobile health (mHealth) research core (mHRC) at a large US academic medical center by leveraging existing research infrastructure. We also discuss the critical role the mHRC played in aiding individual investigators, institutional leaders, and regulatory groups to address system-wide scientific and operational challenges to continuing research in a remote capacity throughout the COVID-19 pandemic.

## Methods

The mHRC at Washington University in St. Louis (WU) was formed in April of 2019. Leaders of the CTSA-supported Washington University Institute of Clinical and Translational Sciences (ICTS) approached the authors, based on the increasing need to support mHealth investigators in navigating institutional regulatory processes, and our experience which included launching the first fully remote clinical trial at the institution [13]. With internal funding from the ICTS, we began the process of developing an institutional mHealth research support function using an adapted agile project management approach to identify barriers to implementing digital research infrastructure and guiding programmatic development.

**Fig. 1.**
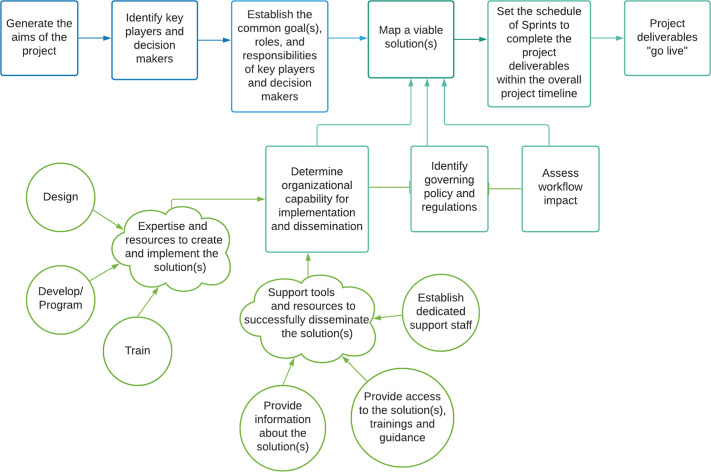
Diagram of the adapted agile project management process applied to implementing digital research methods.

Agile project management accelerates innovation by prioritizing transparency and adaptability, favoring flexibility and iterative project execution over fixed, sequential steps. This approach has been used to promote innovation in research and healthcare through human-centered design, embracing new insights from key stakeholders with early and iterative adaptation. The mHRC adapted an agile project management approach (See Fig. 1) for developing mHealth research capabilities in an academic setting and incorporated the process displayed in Fig. 1 [14–16].

To better understand implementation needs and barriers, we convened an Advisory Committee of investigators and institutional leaders representing clinical informatics, computer science and engineering, implementation science, public health, and clinical disciplines in May 2019. The group established a set of core mHRC offerings to assist investigators, including providing 1) a seminar series to learn about new resources and trends in mHealth research, 2) case consultation to champion peer support and review of ideas, aims pages, manuscripts, and grant proposal, 3) liaison services between groups and University Departments to promote collaborative projects, and 4) online navigation tools to offer guidance for eConsent, virtual sessions, 3rd Party technology partnerships, and computerized systems in FDA regulated trials.

The advisory committee met every 6 weeks throughout the remainder of 2019 to review and address barriers to progress, as well as to discuss new developments and changes. Priorities were focused on developing the four core mHRC service offerings, as follows:

1. **Case Consultations:** Tailored scientific, strategic, technological, and regulatory resources and guidance for emerging researcher needs (e.g., a review of aims page to generate a more detailed research and technology strategy).
2. **Seminar Series:** Regularly occurring seminars featuring virtual talks and panel discussions with leading mHealth research and industry experts.
3. **Liaison Services:** Intermediating among Barnes Jewish Hospital (BJH) and WU departments, centers, and institutions to promote innovation, reduce barriers, improve communication, and streamline process across mHealth projects (e.g., rapidly launching eConsent for COVID-19 studies and generating robust online navigation tools for REDCap eConsent).
4. **Library of Navigation Tools:** A library of online navigation tools (e.g., REDCap eConsent, Telehealth, and Virtual Research Sessions, Partnerships with 3rd Party Technology Vendors) to promote consistency and to prevent future researchers from reinventing the wheel.

In late March 2020, clinical research was completely shut down due to COVID-19. Coincidentally, on the day prior to this announcement, the mHRC had prescheduled one of the regularly occurring seminar series events. As a result, the mHRC devoted a portion of the seminar to a discussion of the imminent shutdown and the need to rapidly shift to fully remote research. This discussion was shared with regulatory leadership, such as the Institutional Review Board (IRB) and Human Research Protection Office (HRPO). To ensure timely and safe mHealth modifications related to COVID-19, the mHRC leadership worked jointly with the Office of Information Security and the IRB to standardize methods for implementing econsent. This collaboration resulted in the mHRC developing online navigation tools for a) creating IRB-approved electronic informed consent using Research Electronic Data Capture (REDCap) and b) conducting study visits using virtual and telehealth technologies. These navigation tools were shared widely in the Institution’s research community. By April 2020, the mHRC added consultation-based services to provide support for investigators transitioning to remote functions.

## Results

### Case Scenario 1: Strengthening an Infrastructure of Support for mHealth Research: Using Existing Digital Resources and Building on Shared Institutional Interests to Rapidly Shift to Remote Clinical Research Methodology during COVID-19

In mid-March 2020, at the beginning of the pandemic, the mHRC anticipated likely “lockdowns” and research shutdowns based on the experience of University of Toronto during the 2003 SARS outbreak in that city (personal communication from Benoit Mulsant, M.D., March 10, 2020). The mHRC recommendations to the university’s IRB included (1) more widespread acceptance of eConsent; (2) rapid turnaround of modifications for remote/contactless research methods; (3) acceptance of protocol deviations for shifts to remote methodology, including investigator reporting of these deviations as a brief statement at the time of renewal (rather than mandating pre-approval of these changes). One week after the research shutdown occurred, the IRB swiftly instituted all recommendations. Research involving human contact was paused, except essential services within certain clinical research. The rapid shift of both COVID-19 and non-COVID-19 research to remote “contactless” methods allowed for continuing existing studies during the pandemic [17,18]. At the same time, WU began initiating studies of patients with COVID-19, who were contagious and/or in quarantine. Investigators immediately sought assistance to incorporate contactless, remote methods to continue their work. The mHRC met this challenge by helping investigators immediately reassess ongoing studies, focusing on the question, “Can the study still recruit and collect participant data?” An example of the mHRC contribution to remote study operations took place when the mHRC worked as a part of the WU COVID-19 Task Force to successfully implement eConsent at WU.

In March 2020, the first project to receive mHRC econsent consultation services swiftly launched a COVID-19 research study and biospecimen bank. The project went from the idea phase to study implementation within 2.5 weeks [19]. The rapid launch of eConsent was largely successful due to a shared common goal, institutional support, project transparency and communication, “pro-active” institutional and study leadership, and WU wide access to REDCap [20] (a highly configurable web-based content management system platform developed by investigators at Vanderbilt University).

A primary goal of this project was to embed the eConsent process into the study site (e.g., emergency department or intensive care unit) workflow while also reducing participant burden and establishing minimal risk of exposure for patients, frontline healthcare workers, and staff engaged in the eConsent process. mHRC consulting services helped achieve this by recommending a verbal (waiver of written) eConsent process, that could be implemented in an efficient and contactless way. During the eConsent process, participants could review and retain a paper copy or be emailed a link to the eConsent for review on their own device or a study-designated tablet in the clinical setting. The person obtaining consent would use the phone to implement the verbal consent process and answer participant questions using REDCap to capture the participant’s response to eConsent. This touchless consent method was approved for this minimal risk study by the IRB and set a precedent for future minimal risk COVID-19 studies using a verbal consent process. The mHRC replicated efforts across 4 additional COVID-19 studies – using either verbal eConsent or eSignature eConsent methods – within 2 months.

Upon the successful launch of eConsent for COVID-19 clinical research, and due to the technical skill required in implementing eSignature eConsent using REDCap, the mHRC assisted research teams in building their eConsent data dictionary using the REDCap online designer tool (see Table 1) [21]. The mHRC also helped to establish a WU eConsent Workgroup involving key institutional and regulatory leadership. The initial goals of the eConsent Workgroup were to establish an eConsent process for all research at WU (including non-COVID-19 research) with broad regulatory approval and to improve upon existing guidance (e.g., the mHRC REDCap eConsent Navigation Tool) to instruct research teams clearly and concisely in developing and implementing approved eConsent processes. Navigating REDCap can be straightforward for developing study electronic case report forms (eCRF), deploying digital assessments and implementation of verbal eConsent that does not require a participant signature (such as in minimal risk studies). However, the process becomes more nuanced and technical with secure and encrypted emails, eConsent versioning and when an eSignature is required (e.g., to be adherent to regulatory guidelines and university policy). For example, institutions adhering to the ICH Regulations [22] will require a signature from both the participant and the person obtaining consent. There may also be additional university policy that indicates the order of signing the eConsent, and the mHRC REDCap eConsent Navigation Tool offers a section with advanced instructions for developing an eConsent that includes the WU approved signatory process (i.e., the participant signs first and the person obtaining consent signs thereafter). The mHRC eConsent navigation tool also links to a “REDCap in a Flash” [23] webinar tutorial offered by the Institution that leads research teams through the process of implementing the mHRC eConsent Navigation Tool.

**Table 1.** Example of utilizing adapted agile project management approach for developing mHealth research capabilities in academic settings

Implementation
**First Aim of the project:** Rapidly launch COVID-19 research using eConsent
**Second Aim of the project:** WU to build an infrastructure of support for eConsent and remote “contactless” methodology in research
**Key players:** mHRC; The WUSM Becker Medical Library “REDCap in a Flash” video tutorials
**Key decision-makers:** WU regulatory and management leadership; Study Principal Investigators
**The common goal was to promote successful launch and continuation of research through the implementation of eConsent**. The mHRC was to build REDCap eConsent for COVID-19 research teams and provide guidance and support tools with institutional oversight from WU regulatory and management leadership
**The viable platform solution** was to implement eConsent using REDCap due to access, low to no cost to study teams, and familiarity with the platform at WU
**The mHRC mapped out the REDCap Academia technical specifications for the platform to be used for eConsent:** The mHRC determined a high level of familiarity and ease of use with REDCap would be required to implement eConsent in accordance with WU policy and ICH regulations
**Organizational capability to disseminate and implement change:** Beyond the mHRC building the eConsent into the study workflow for COVID-19 research, there was no training in place for research teams to implement eConsent successfully on their own, which could have led to disruptions in workflow. Therefore, the mHRC helped form the WU eConsent Workgroup and focused on creating a more robust online REDCap eConsent navigation tool and partnered with the WUSM Becker Medical Library “REDCap in a Flash” service to generate video tutorials for mass dissemination of eConsent at WU [21,23]

WU = Washington University in St. Louis; mHRC = mobile health (mHealth) Research Core; REDCap = Research Electronic Data Capture; eConsent = Electronic Consent; ICH = International Council for Harmonisation of Technical Requirement for Pharmaceuticals for Human Use; WUSM = Washington University School of Medicine.

A lesson learned while implementing digital research methods at an Academic Medical Center during COVID-19 is to acknowledge that context shapes the nature and course of digital transformation. Particularly in public health disaster scenarios, there may not be time or resources available to adopt the ideal long-term solution. Change initiatives at the organizational level, particularly those involving technology, often fail without carefully executed change management plans [24]. However, disasters can open windows of opportunity for innovation and illustrates how an inevitable change – transitioning to fully remote clinical research – was accelerated by pandemic circumstances. While this provided an important opportunity, it also limited our options to those available (and affordable) at the time. In our case, the pandemic created an urgency and an openness to digital transformation that might not have otherwise existed. The next challenge will be transitioning to more efficient systems and processes while continuing to adjust to the new and swiftly evolving normal in public health. The mHRC will continue communication with executive management and decision-makers about relevant limitations of an identified solution and is working toward incorporating a process of continuous evaluation to assess the impact of the currently implemented solution (REDCap eConsent), the evolution of the business need, user feedback, available resourcing, allocated funding, and potential alternative solutions.

In summary, as part of the eConsent Workgroup, the mHRC continues to invest in the following areas of improvement and expansion in the implementation of eConsent at the University:

1. **A more robust REDCap eConsent Navigation Tool:** The mHRC REDCap eConsent Navigation Tool was approved by the IRB and mitigated confusion and errors across study teams. The mHRC continues to develop improved iterations of the REDCap eConsent Navigation Tool and has partnered with The WUSM Becker Medical Library to create instructional “REDCap in a Flash” video tutorials.
2. **Integrated navigation tools and templates:** The mHRC continues to work with the WU eConsent Workgroup to create an eConsent template that will be embedded within REDCap. This embedded template will provide study teams the option to “turn on” eConsent within REDCap and reduces the amount of work-study teams will spend building their project in REDCap.
3. **Specialized eConsent case consultations:** For studies needing assistance above and beyond the online Navigation Tools and video tutorials, the mHRC offered direction by way of 30-minute case consultations focused on implementing eConsent.
4. **eConsent liaison services**: As part of the WU eConsent Workgroup, the mHRC is working with the Center for Clinical Studies Trial-CARE Unit to roll out a University supported eConsent and EDC suite of services for training, standard operating procedures, and validation of computerized systems used in FDA CFR 21 Part 11 compliant regulated research [25].

### Case Scenario 2: Continuing Research Uninterrupted During the Pandemic: Working with 3rd Party Vendors

Most academic and medical research institutions respect the autonomy of investigators and may not provide the authority to require investigators use a particular vendor. This places the responsibility on the investigator when identifying and vetting technology vendors and associated products and costs. In the case of using an existing Software as a Service (SaaS) product, costs would be invested in licensing the product for use. SaaS products may be a reasonable option for many investigators with the goal of implementing research using technology.

Third-party SaaS vendors may offer a variety of options from highly configurable platforms to more “off the shelf” (static) platforms. The mHRC developed the Technology Partnerships Navigation Tool to help investigators navigate conversations with 3rd party vendors to determine a partnership of good fit, including but not limited to intellectual property rights, regulatory compliance, and cost considerations.

**Fig. 2.**
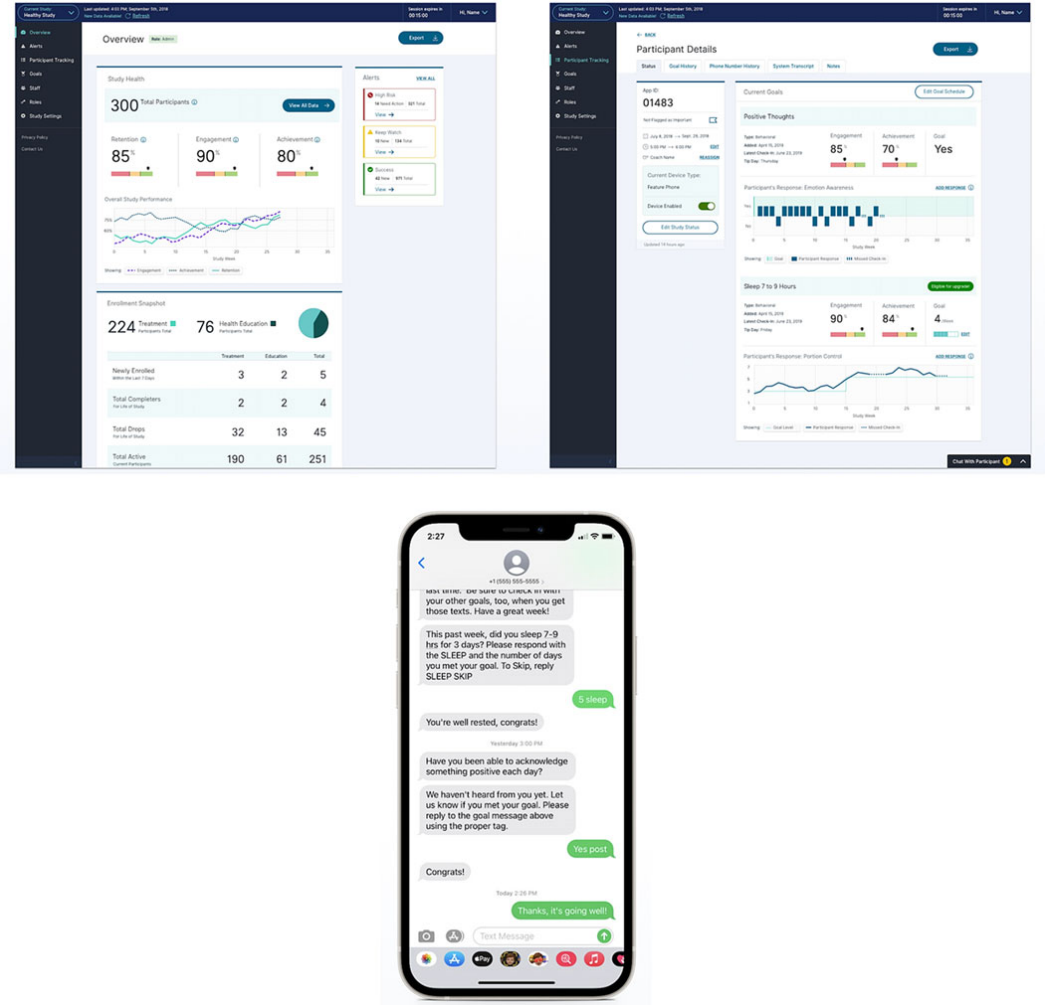
Image of healthyMedium Content Management System (healthyCMS) and SMS intervention as used in the Healthy Bodies Healthy Minds study.

Healthy Bodies Healthy Minds (HBHM) is a multisite pilot clinical trial utilizing an adapted Interactive Obesity Treatment Approach (iOTA) [26,27] for obesity prevention in young adults with severe mental illness and overweight or early-stage obesity [26]. HBHM is one example of a project that required the engagement of a 3rd party tech vendor with a configurable SaaS content management system. The study investigator selected to work with the 3rd Party technology vendor happyMedium | healthyMedium, LLC [28] to integrate the adapted iOTA SMS text message intervention with the vendor’s proprietary healthy Medium Content Management System (healthyCMS, Fig. 2).

The mHRC helped the study investigator navigate the partnership with the 3^rd^ Party Technology vendor and utilized an adapted agile project management approach to help the study successfully launch and continue operations uninterrupted during the pandemic (See Table 2).

**Table 2.** Example of utilizing adapted agile project management approach to successfully launch a pilot treatment adaptation study in a fully remote multisite clinical trial

Implementation
**First Aim of the project:** Rapidly build the capacity for fully remote study “start-up” of remote clinical sites in a mHealth obesity prevention and weight loss study for young adults with severe mental illness. Priority areas included developing an online, interactive training for the clinical (non-research) staff (e.g., intervention delivery, data collection, and use of the healthyCMS)
**Second Aim of the project:** Using evidence-based frameworks for digital treatment adaptation [29,30], the study aimed to make incremental adaptations to the HBHM intervention and the configuration of the healthyCMS during a multisite pilot study evaluating both intervention implementation in real-world settings, as well as to evaluate the effectiveness of the intervention
**Key players and decision-makers:** Study Principal Investigator; mHRC; happyMedium | healthyMedium, LLC
**The common goal was to successfully launch a pilot treatment adaptation study in a fully remote multisite clinical trial**. The mHRC helped the study team to identify existing resources for eTraining development and for incorporating eConsent and telehealth sessions to successfully launch a fully remote study early in the pandemic
**The mHRC provided troubleshooting to leverage an existing product (e.g., healthy Content Management System or healthyCMS) for study onboarding, ongoing training, and intervention delivery.** The mHRC helped the study team incorporate existing high-quality healthyCMS video tutorials into the remote intervention training. Feedback on the training, ease of use of the healthyCMS were collected from the study team to influence future training and treatment adaptation
**Organizational capability to disseminate and implement change:** happyMedium | healthyMedium offered a technology package to implement the healthyCMS in a HIPAA compliant manner with intuitive user interface and user experience design and tutorials for utilizing the healthyCMS and customized the CMS with study-specific tools and tips. Brief and engaging screen-recorded video tutorials were developed based on the CMS model, and then disseminated to the study team along with access to a user acceptance testing (UAT) version of the CMS to practice in prior to seeing their first study participant

healthyCMS = healthyMedium Content Management System; HBHM = Healthy Bodies Healthy Minds; mHRC = Mobile Health (mHealth) Research Core; LLC = Limited Liability Company; HIPAA = Health Insurance Portability and Accountability Act; CMS = Content Management System; UAT = User Acceptance Testing.

The HBHM study launch coincided with the beginning of the pandemic, which necessitated the shift to completely remote study functions. Thus, an electronic clinical trial management system was needed for overall study management of remote clinical sites, including participant tracking and data management.

The proprietary healthyMedium content management system (healthyCMS, Fig. 2) was used to deliver intervention-specific SMS messaging and collect precise participant-level data, including ecological needs assessments [31] (i.e., real-time assessments of user needs and satisfaction with the intervention). The healthyCMS tracked participant and site-level engagement and retention metrics and provided a tailored integration with Twilio.com to facilitate the HBHM SMS text messaging treatment adaptation of the parent study’s SMS messaging content [26]. The mHRC facilitated communication between the investigators and the tech vendor, providing support throughout all phases of the project. The project start-up objectives additionally included 1) finalize contract and scope of work, with consideration for longer-term issues such as ongoing data management and system upkeep, 2) assist with obtaining information security and IRB approvals for a multisite clinical trial utilizing an existing CMS, and 3) aid in crafting an adaptation plan adherent to original study budget and aims, allowing room for flexibility in implementation. For example, the mHRC worked with the study team and 3rd party vendor to determine the SMS intervention would be the most economical starting place for the HBHM feasibility study, given the SMS intervention logic was largely established by the parent study. However, the plan was also set in place for HBHM to grow into a web-based intervention with modified Twilio SMS messaging and server-based notifications. Web-based applications can be an economical choice for many studies because one code source can be used and the work product would be accessible across multiple device types – computer, tablet, iOS, and Android. However, web-based applications are dependent upon Internet connection and may not function when there is not a connection. Therefore, web-based applications may not be appropriate to use in all study types (e.g., where response times are data outcomes or for studies in remote areas without reliable Internet connection), and special consideration must be given to how the data will be securely retained and transferred in case there is disruption to internet service. The liaison services of the mHRC were also critical in helping the investigator map implementation solutions utilizing existing accessible technology embedded in the project workflow, Zoom, and the healthyCMS.

The healthyCMS integration with SMS text messaging and the use of Zoom for study trainings and virtual study visits enabled the study to launch in a fully remote capacity and to continue uninterrupted amid the COVID-19 pandemic. Although no changes to the original research protocol were required to shift to fully remote study procedures, this study benefitted from mHRC assistance in building a partnership with a technology vendor that provided the healthyCMS Software as a Service (SaaS) product, making use of existing digital resources for eConsent (see Case 1), secure virtual video study visits and remotely training study interventionists.

## Discussion/Summary

The initial mHRC core offerings were designed to achieve critical impacts for WU research, including but not limited to the following: (1) educate investigators on how to best implement mHealth tools and concepts in their research, (2) help research studies and research teams operate in a remote capacity, and (3) promote an infrastructure of support for investigators employing mHealth tools or testing mHealth interventions in their research. These critical impacts were achieved by the mHRC through the implementation of the mHRC core offerings, and implementation of an adapted agile project management approach to ensure investigators could rapidly shift to fully remote study operations, continue ongoing studies and start newly funded studies during the pandemic.

Reflecting the academic nature of the work, we published preliminary observations and recommendations from the mHRC’s work, including continuing and even expanding clinical research via mHealth during the COVID-19 pandemic [18,32]. These recommendations, which remain relevant more than a year later, included implementing technology to reduce the need for face-to-face contact with participants. Researchers can minimize face-to-face contact with participants by using digital tools, such as electronic informed consent and digital HIPAA compliant SMS text messaging, mobile app or web assessments, and virtual visits.

The mHRC will continue facilitating and accelerating new and ongoing research across clinical areas and departments at WU. To foster inter-institutional collaboration among mHealth researchers at CTSA institutions, a future direction of the mHRC is to generate a resource page on our website that lists mHealth researchers and their associated area of research and institution. Even as the pandemic recedes in the USA, remote research methodology is here to stay. We hope this description of the mHRC experience can serve as a roadmap for institutions facing what are likely to be universal needs at academic medical institutions: go-to resources for researchers across clinical areas looking to utilize digital tools in remote study methodology, the reduction of technical and regulatory delays, and the sharing of accumulating knowledge and experience within and between CTSA-supported academic institutions to achieve research aims and collaboration among mHealth researchers.
